# Biomechanics and finite element analysis comparing posterior T-plates with LCP for fixation of posterolateral tibial plate fractures

**DOI:** 10.3389/fbioe.2023.1286993

**Published:** 2023-12-07

**Authors:** Zhenghui Hu, Weizhi Ren, Jian Peng, Zenghui Gu, Chenying Wu, Weicheng Wu, Wen Zhang, Wei Xu, Liubing Li

**Affiliations:** ^1^ Department of Orthopedics, The Second Affiliated Hospital of Soochow University, Suzhou, China; ^2^ Orthopedic Institute of Soochow University, Suzhou, China

**Keywords:** posterolateral tibial plateau fracture, biomechanical research, retention strength, finite element analysis, lateral approach

## Abstract

**Objective:** The treatment for posterolateral tibial plateau fractures (PTPF) have been subjects of controversy. We conducted a study to improve the fixation of PTPF through a lateral approach.

**Methods:** We utilized 40 synthetic tibias and categorized the fracture models into five groups based on the locking compression plate (LCP) and T-distal radius plate (TPP) via various forms of fixation with screws through the posterolateral (PL) fracture fragments. I: Two-screw fixation using two locking screws (LPTL). Ⅱ: Two-screw fixation with both variable angle locking screws (LPTV). Ⅲ: One-screw fixation with one locking screw (LPOL). Ⅳ: One-screw fixation with one locking screw and two anteroposterior lag screws (LPOLTL). Ⅴ: a distal radius plate with three locking screws (TPP). Biomechanical tests were conducted to observe the axial compression displacement of the PL fracture fragments at force levels of 250 N, 500 N, and 750 N, as well as to determine the failure load and the axial stiffness for each respective group.

**Results:** Under a 750 N load condition, the displacements within the five experimental groups exhibited the following trend: Ⅴ < Ⅱ < Ⅰ< Ⅳ < Ⅲ. However, there were no significant differences between Group V and Group II, Group I and Group IV (*p* > 0.05), and only Group Ⅲ demonstrated a displacement exceeding 3 mm. The failure load and the axial stiffness exhibited the same trend. Conversely, statistical significance was identified among the remaining group compared with Group Ⅲ (*p* < 0.05). Regarding the finite element analysis, the maximum displacements for the five models under the load of 750 N exhibited the following trend: Ⅴ < Ⅱ < Ⅰ< Ⅳ < Ⅲ. The following trends were observed in maximum von Mises stresses for these models under the load of 750 N: Ⅴ < Ⅱ < Ⅳ< Ⅰ < Ⅲ.

**Conclusion:** It is crucial to address the inadequate mechanical strength associated with single screw fixation of LCP for fixing PL fractures in a clinical setting. The biomechanical strength of two-screw fixation surpasses that of single-screw fixation. Introducing variable-angle screws can further enhance the fixation range. Furthermore, the addition of two lag screws threaded from anterior to posterior can compensate the mechanical stability, when PL fracture is fixed with single screw in clinic.

## 1 Introduction

The tibial plateau confers essential stability to the knee joint while also functioning as a pivotal weight-bearing interface (Schatzker and Kfuri, 2022). Given the widespread adoption of computed tomography (CT), posterolateral tibial plateau fractures (PTPF), either in isolation or concomitant with other columnar fractures, have emerged as more prevalent than previously documented in the literature (Shen et al., 2019; Chen et al., 2020; Cai et al., 2021). It is worth noting that articular surfaces >2 mm and inversion deformities >4° contribute to an elevated risk of osteoarthritis (Honkonen, 1994; Rademakers et al., 2007; Parkkinen et al., 2014; Shen et al., 2019). Hence, it is imperative to underscore the significance of proper treatment of PTPF.

PTPF constitute 8%–15% of all plateau fractures, resulting from a combination of valgus and axial stresses during knee flexion (Higgins et al., 2009). The unique injury mechanism often leads to predominantly posterior and lateral displacement in terms of fracture displacement trends (Zhang et al., 2020). As a result, surgical access has primarily been explored through lateral or posterior approaches. Although the posterior approach and implant fixation offers biomechanical benefits, it comes with a heightened risk of iatrogenic injury and limited exposure of the fracture site (Kim et al., 2018; Cai et al., 2021; Durigan et al., 2023). On the other hand, the lateral approach stands as one of the most prevalent surgical options embraced by a majority of surgeons, given its lower risk of vascular and nerve injuries (Cho et al., 2017; Sun et al., 2018; Hu et al., 2020).

Given these considerations, the question arises: Can the lateral approach be a viable option for achieving satisfactory fixation strength in cases of PTPF?

It was established that the biomechanical strength conferred by a solitary screw for posterolateral (PL) fracture fragment fixation with locking compression plate (LCP) does not align with the physiological demands of the human body (Zhang et al., 2012). Building upon previous work (Hu et al., 2020; Zhang et al., 2020) in biomechanical assessment of LCP-immobilized PL fractures, our study delved into the disparities in mechanical strength stemming from differences in the number of screws fixation in LCP transverse-arm to stabilize the PL fracture fragment through the lateral approach.

## 2 Materials and methods

The current fracture model was established upon the work of Sohn et al. (Sohn et al., 2015). Our synthetic bone model (Synbone, type 1110. SYNBONE AG, Kulai, Johor, Malaysia) was composed of rigid foam, infused with cancellous bone material to replicate the properties of a normal tibia. The procurement was carried out from a manufacturer that ensured uniformity in terms of material composition, structure, and attributes. Following the axial compression of the PL fracture fragment using a custom-designed T-shaped applicator, the artificial tibia was securely positioned within an embedding container containing custom self-coagulating dentine powder and dentine water. To attain satisfactory solidification of the dentine powder, the cooling period was limited to 60 min. Subsequent to fixation, specialized biomechanical testing was conducted employing dedicated equipment.

In our experiment, a total of 40 synthetic tibiae were utilized, distributed across five groups with an average of eight specimens per group. These tibiae were employed to simulate a posterior lateral split fracture model. For lateral fixation, a 3.5-mm LCP was employed, while posterior fixation was achieved using a distal radius support plate. To secure the fracture fragment, a combination of locking screws with varying lengths, variable-angle locking screws, and lag screws were utilized.

### 2.1 Posterolateral fracture model Construction

**FIGURE 1:**
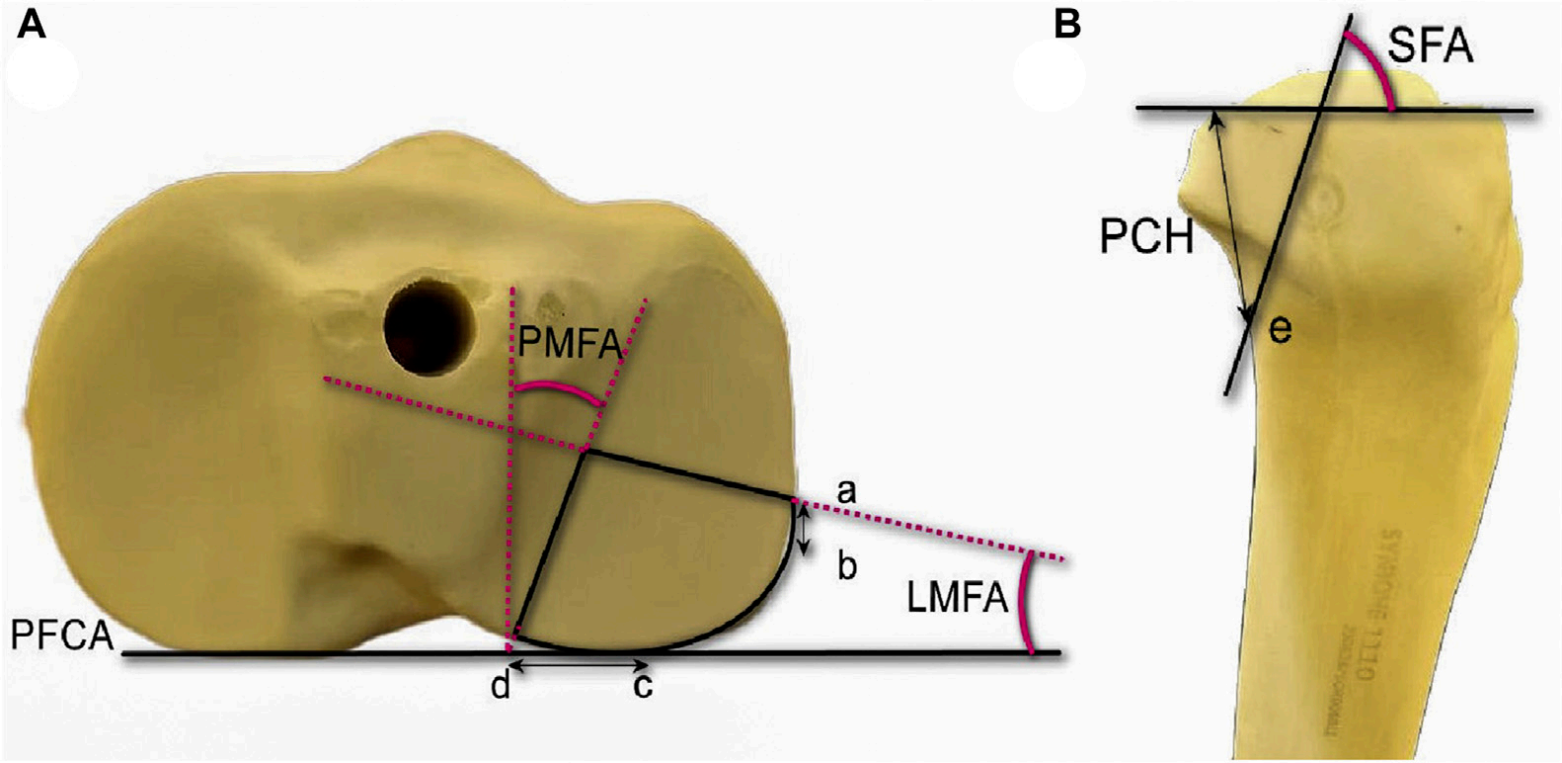
Cranial and lateral views of the posterolateral (PL) split fracture model of tibial plateau. **(A)** Cranial view. PFCA, posterior femoral condyle axis; point a, lateral exit point of the PL fracture; point b, anterior edge of the articular facet of fibular head; point c, medial edge of the articular facet of fibular head; point d, posterior exit point of the PL fracture; angle LMFA, angle between the lateral fracture line of the PL fragment and the PFCA; angle PMFA, angle between the medial l fracture line of the PL fragment and the line perpendicular to the PFCA. **(B)** Lateral view. angle SFA, angle between the joint line of the PL fragment with the coronal fracture line; point e, exit point below PL fracture.

The PL fracture model is established with the details of the model outlined in Figure 1. The LAPD is the vertical distance from the lateral exit point of the PTPF to the anterior cortex of the fibular head. The anterior edge of the fibular head was designated as point b with an Line ab dimension of 10 mm to ascertain the exit position of the lateral margin of the fracture line, identified as point a. Subsequently, an osteotomy was executed with a lateral margin fracture angle (LMFA) of 13°. The PHD is the horizontal distance between the medial cortex of the fibular head and the posterior exit point of the posterolateral column fracture. Posteriorly, the medial margin of the fibular head was utilized as point c, with a Line cd measure of 23 mm to determine the exit location of the posterior medial margin, denoted as point d, along the fracture line. The osteotomy was carried out with a posterior margin fracture angle (PMFA) of 20°. The exit point e of the lower fracture line was established based on the coordinates of point a, point d, a sagittal fracture angle (SFA) of 78°, and a posterior coronal height (PCH) value of 31 mm. Subsequently, an osteotomy was executed. Our modeling referenced previous studies (Sun et al., 2018; Zhang et al., 2020; Ren et al., 2022).

Osteotomies were executed using a thin-knife chainsaw, and geometric measurements were conducted utilizing AutoCAD software (AutoCAD, 2020; Autodesk, San Rafael, CA, United States). All geometric measurements and preparations were carried out under the supervision of an experienced surgeon. To enhance uniformity, a skilled swing saw operator was chosen after undergoing training on 30 artificial tibiae. This approach aimed to ensure consistent size and shape of the fracture fragment in each instance.

### 2.2 Grouping of fixation models of PL fracture

**FIGURE 2:**
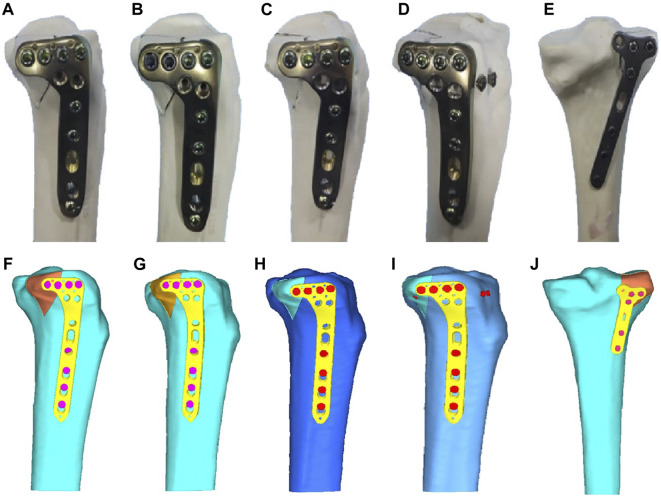
Five different internal fixation models of the posterolateral (PL) fracture. **(A)** Fixation with two conventional locking screws in the transverse arm of the LCP. **(B)** Fixation with two variable-angle locking screws in the transverse arm of the LCP. **(C)** Fixation with one locking screw in the transverse arm of the LCP. **(D)** Fixation with one locking screw in the transverse arm of the LCP plus two anterior and posterior lag screws. **(E)** Posterior support with a T-shaped distal radius plate fixation. **(F)** Fixed by two locking screws of the LCP of the finite element model. **(G)** Fixed by two variable angle locking screws of the LCP of the finite element model. **(H)** Fixed by one locking screw of the LCP of the finite element model. **(I)** Fixed by one locking screw with two-antero-posterior lag screws of the LCP of the finite element model. **(J)** Fixed by posteriorly supported T-shaped distal radius plate of the finite element model.

The created split fracture model was meticulously repositioned and subsequently secured with two Kirschner pins, extending from the posterior-lateral to posterior-medial aspect, to ensure precise anatomical realignment. The plate was affixed to the tibial cortex, aligning the upper margin of the plate with the articular surface of the tibia, while the longitudinal arm of the plate maintained a parallel orientation to the tibia’s longitudinal axis. Five groups of plate fixation strategies were employed in constructing the fracture model, as delineated below. Group Ⅰ: the LCP was postposition and the anterior edge of the penultimate locking screw of the plate was positioned lateral exit point of the PL fracture, thereby permitting PL fracture fragment fixation with two conventional locking screws (LPTL). Group Ⅱ: the plate position was similar to Group Ⅰ. Differently, the last two locking screws were substituted with variable angle locking screws (10° posterior offset, LPTV). Group Ⅲ: the LCP was not postposition and PL fracture fragment was fixed with only one locking screws (LPOL). Group Ⅳ: the plate position was similar to Group Ⅲ, PL fracture allowing for the secure passage of a complete screw. Additionally, two lag screws were introduced from the anterior to posterior direction, fixing PL fracture beneath the locking screw (LPOLTL). Group Ⅴ: the T-type distal radius plate was situated to rear of the PL fracture, ensuring two screws of the transverse arm plus one screw of the longitudinal arm effectively fixing the fracture fragment (TPP) (Shen et al., 2019) (Figures 2A–E).

All the materials mentioned above were sourced from the same manufacturer to ensure uniform material properties. The creation of fracture models was carried out consistently by a single experienced surgeon to replicate fracture reduction and fixation. It is important to note that factors influencing knee joint pressure, such as ligaments, muscles, and surrounding soft tissues, were not taken into account in this study.

### 2.3 Biomechanical testing and finite element analysis

#### 2.3.1 Biomechanical testing

**FIGURE 3:**
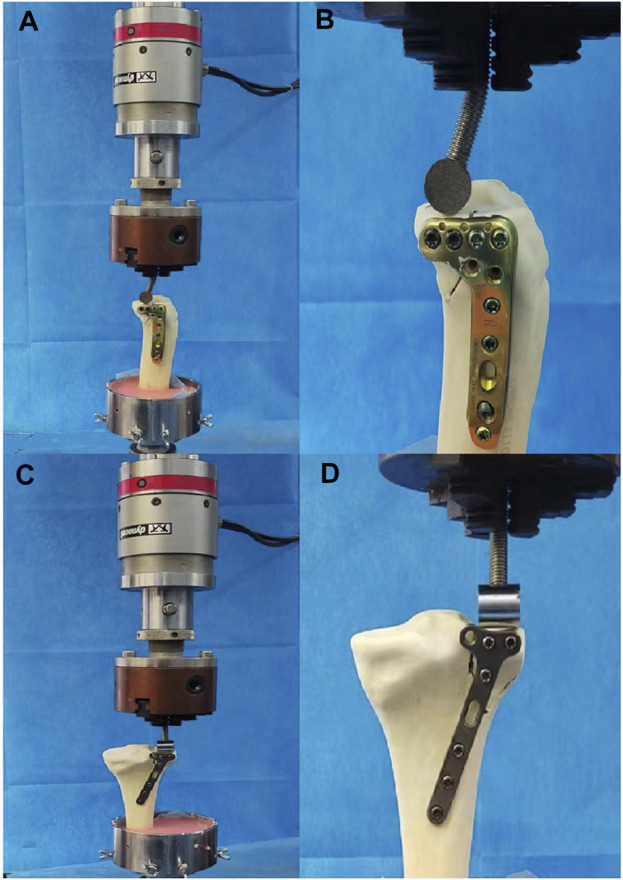
Positioning of different fixation model of PL fracture within the machine. **(A)** Fixation of the locking compression plate at the lateral side. **(B)** Local magnification of **(A)** showing biomechanical test. **(C)** Fixation of T-shaped distal radius plate at the posterior side. **(D)** Local magnification of **(C)** showing biomechanical test.

Each model was positioned on a material testing machine (E10000 Linear-Torsion All-Electric Dynamic Test Instrument, InstronE10000, Instron Corporation Norwood, MA, United States) for testing (Figure 3). To account for the femur’s compression against the plateau during body flexion, a T-shaped applicator (designed to mimic shear stress) was customized to apply compression to the posterolateral fracture fragment (Feng et al., 2021).

Considering that the biomechanical load experienced by a normal adult knee is approximately 2–3 times the body weight, with the lateral plateau bearing 45% of this load (Ogaya et al., 2014), we aimed to replicate the load in our experiments. For instance, with a 60 kg adult body weight, the pressure on the lateral platform approximates 250, 500, and 750 N for 1–3 times the body weight. Therefore, axial loads of 250, 500, and 750 N were selected to simulate the lateral platform loads in our experiments. These loads were applied at a rate of 10N/s to each fracture model after mounting.

During testing, axial displacement was continuously monitored from the initial position to peak load using axial displacement software, integrated with Bluehill software (Instron, Norwood, MA, United States). Load-displacement curves were generated for each fracture model. Failure load was defined as the vertical displacement of the posterior lateral fracture fragments up to 3 mm. The maximum peak load was limited to 750 N or the load corresponding to a deformation of 3 mm. As a result, we evaluated biomechanical stability using displacements at 250, 500, 750 N, and failure load levels.

### 2.4 Statistical analysis

We employed IBM SPSS Statistics 27 for statistical analysis to assess the vertical displacements of the five fracture models under distinct loading conditions, along with the failure loads, treated as measures conforming to a normal distribution. To conduct comparisons, we utilized one-way ANOVA, with a significance level set at *p* < 0.05 to establish statistical significance.

### 2.5 Finite element analysis

With the informed consent obtained, we enrolled a healthy adult male volunteer, aged 30, devoid of knee ailments and major systemic health conditions, to participate in the study. Employing a 64-row multislice spiral CT scanner, scans were conducted from the knee to the ankle, maintaining a slice spacing of 0.625 mm. The acquired CT images were stored in DICOM format within Mimics software (version 19.0, Materialise, Leuven, Belgium). Subsequently, a three-dimensional model of the tibia was formulated, leveraging the tissue’s grayscale values and region segmentation. This preliminary model underwent refinement in Geomagic Studio (version 12, Geomagic, NC State, United States) through a smoothing process, rectifying the three-dimensional model’s surface. The various segments of the finite element model were then imported into Hypermesh software (version 2017, Altair, Inc., United States), a meshing tool for finite element analysis. Meshing was performed employing quadratic tetrahedral elements (Solid187) to ensure optimal discretization. The tibia was characterized as isotropic, linear elastic, and homogeneous. Each model was constructed using quadratic tetrahedron elements ranging in size from 0.5 to 1.0 mm. A convergence test was executed across all models, ensuring that the maximum change remained below 1%. The material properties were validated based on the previous work (Huang et al., 2019). The three-dimensional model of the plate and screws was developed in compliance with the manufacturer’s specifications using computer-aided design software Creo Parametric (PTC, Inc., United States). All interactions between fracture fragments and implants were modeled as frictional contact, with a coefficient of friction of 0.4 assigned to replicate the conditions (Rancourt et al., 1990). The tibia model was integrated into Geomagic Studio software (3D Systems Inc., Rock Hill, SC, United States).

For the internal fixation models of PL tibial plateau fractures, the tibia model was combined with internal fixations using Creo Parametric software, utilizing relative data. It is worth noting that our finite element analysis models had been validated previously (Ren et al., 2022). Five models of fracture fixation were same with the groups of biomechanical testing. That was Ⅰ: Group LPTL; Ⅱ: Group LPTV; Ⅲ: Group LPOL; Ⅳ: Group LPOLTL; and Ⅴ: Group TPP (Figures 2F–J).

Axial compression of the PL split fracture fragment was executed utilizing three distinct axial loading conditions: 250, 500, and 750 N, with the load applied perpendicular to the tibial plateau. The Young’s modulus (MPa) and Poisson’s ratio used for the finite element analysis were outlined in Table 1 (Qiu et al., 2011; Anwar et al., 2017; Huang et al., 2019). Meanwhile, Table 2 provided insight into the node and element counts for each group of models. The analysis of five fixation models was conducted using ANSYS Mechanical APDL 19.0 software (ANSYS, Inc., United States).

**TABLE 1:** The Young’s modulus (MPa) and Poisson’s ratio.

Material	Young’s modulus (MPa)	Poisson’s ratio
Plate	110000	0.3
Screw	110000	0.3
Cortical bone	14000	0.3
Cancellous bone	700	0.3

**TABLE 2:** Number of elements in five groups of models.

Model	Nodes	Elements
LPTL	823586	539115
LPTV	813802	531882
LPOL	902541	552457
LPOLTL	863564	562391
TPP	781469	514402

## 3 Results

### 3.1 Biomechanical testing of five fixation model for PTPF

**FIGURE 4:**
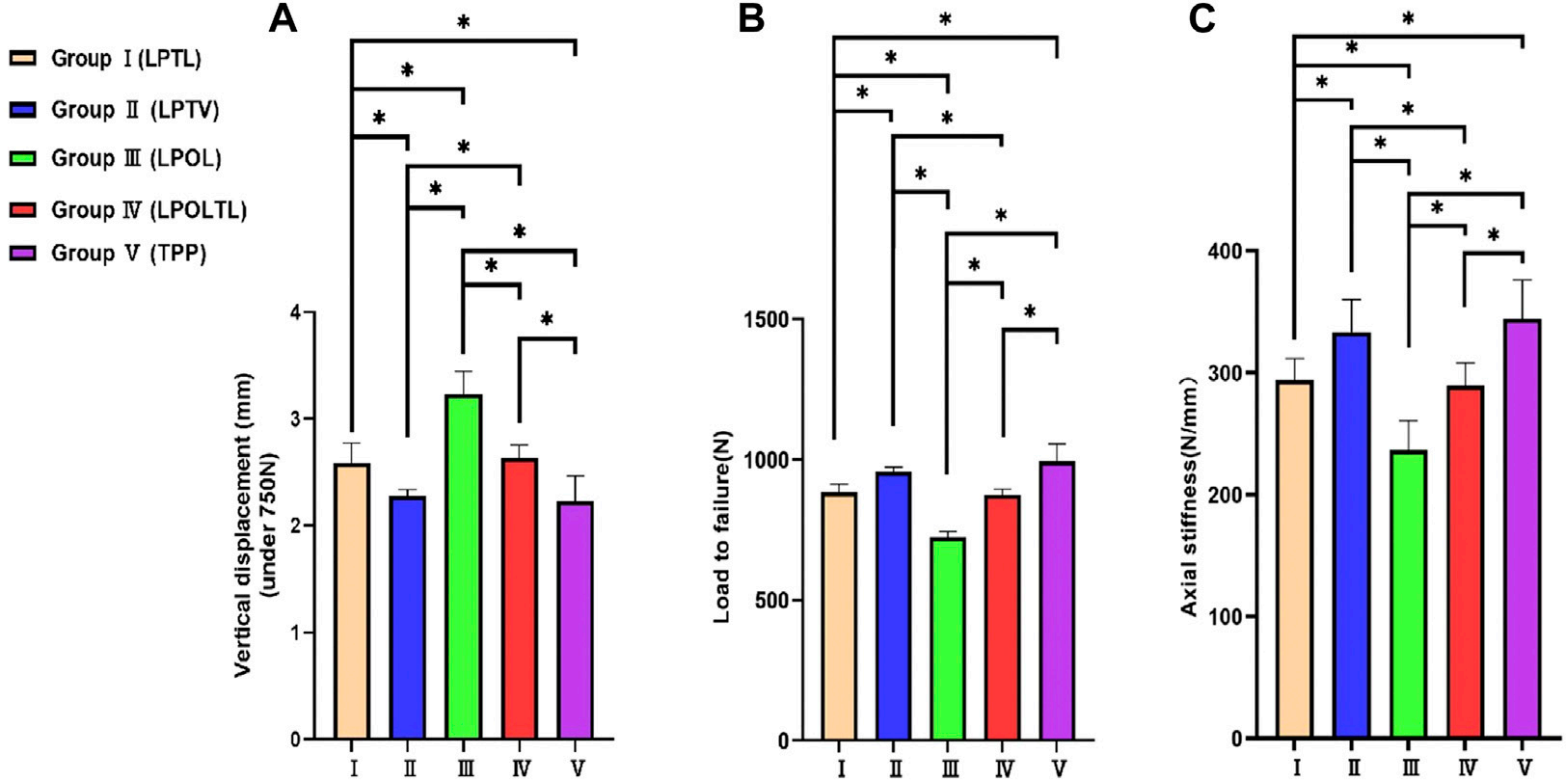
Synopsis of statistical analyses across the five model groups. **(A)** The displacement of the posterolateral fracture fragment under 750N axial loads. **(B)** The Load to failure for five groups of models. **(C)** The Axial stiffness for five groups of models. The bars indicated the mean, and the error bars indicated the standard deviation. *p < 0.05.

Biomechanical Testing of five fixation model was performed including the vertical displacement across three distinct axial loads, failure loads and axial stiffness (Table 3). The trends in displacement were consistent across all five fracture fixation models, ranging from 250 to 750 N. The findings indicate that Group Ⅴ exhibited the least displacement, while Group Ⅲ demonstrates the highest degree of displacement. The disparity in displacements between Group Ⅴ and Group Ⅱ did not attain statistical significance (*p* > 0.05). The distinction in displacements between Group Ⅰ and Group Ⅳ did not achieve statistical significance (*p* > 0.05). Significant disparities in displacements were identified among the remaining four groups when compared to Group Ⅲ (*p* < 0.05) (Figure 4A).

**TABLE 3:** Vertical displacement, failure loads and axial stiffnesses of five sets of fracture models under three different loads.

Group	Vertical displacement (mm) (mean ± SD, mm)			Load to failure (mean ± SD, N)	Axial stiffness (mean ± SD, N/mm)
	250N	500N	750N		
LPTL	0.86 ± 0.07	1.70 ± 0.08	2.60 ± 0.18	885.40 ± 27.72	295.10 ± 9.24
LPTV	0.73 ± 0.09	1.50 ± 0.13	2.28 ± 0.06	957.50 ± 16.31	319.20 ± 5.44
LPOL	1.13 ± 0.18	2.06 ± 0.20	3.24 ± 0.22	723.40 ± 20.55	241.10 ± 6.85
LPOLTL	0.89 ± 0.11	1.70 ± 0.09	2.64 ± 0.12	874.50 ± 21.45	291.50 ± 7.15
TPP	0.70 ± 0.07	1.45 ± 0.14	2.24 ± 0.23	994.20 ± 61.41	331.40 ± 20.47

The failure load and axial stiffness exhibit a comparable trend to the displacement. Figures 4B, C illustrate the failure loads and axial stiffness values across the five model groups, along with the associated statistical variances. Group Ⅴ exhibited a maximum failure load and axial stiffness, while Group Ⅲ the lowest values. The difference in failure load and axial stiffness between Group Ⅴ and Group Ⅱ did not attain statistical significance (*p* > 0.05). Likewise, the distinction between Group Ⅰ and Group Ⅳ did not achieve statistical significance (*p* > 0.05). Significant disparities were identified among the remaining four groups compared to Group Ⅲ (*p* < 0.05).

### 3.2 Finite element analysis results test

**FIGURE 5:**
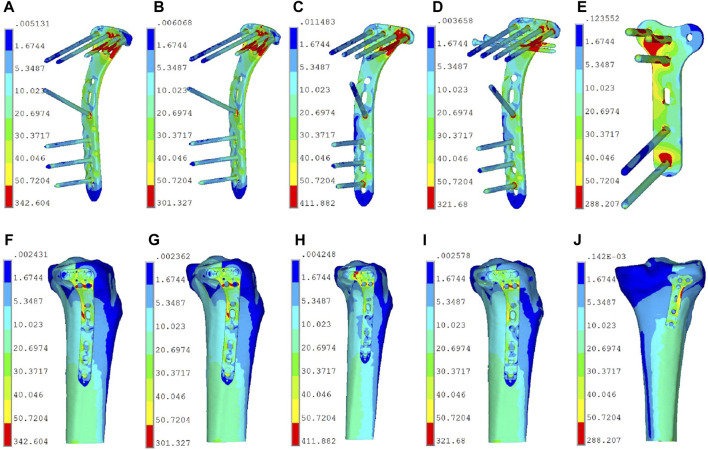
The von Mises stress distribution of among five groups of internal fixation devices and both bone and internal fixation. **(A)** Group Ⅰ (LPTL) internal fixation stresses. **(B)** Group Ⅱ (LPTV) internal fixation stresses. **(C)** Group Ⅲ (LPOL) internal fixation stresses. **(D)** Group Ⅳ (LPOLTL) internal fixation stresses. **(E)** Group Ⅴ (TPP) internal fixation stresses. **(F)** Group Ⅰ (LPTL) both bone and internal fixation stresses. **(G)** Group Ⅱ (LPTV) both bone and internal fixation stresses. **(H)** Group Ⅲ (LPOL) both bone and internal fixation stresses. **(I)** Group Ⅳ (LPOLTL) both bone and internal fixation stresses. **(J)** Group Ⅴ (TPP) both bone and internal fixation stresses.

The displacements and stresses observed in the five fixation groups of PL split fractures under loads ranging from 250 to 750 N displayed in Table 4, and the von Mises stress distributions were detailed in Table 5. The finite element analysis showed the displacements at 750 N were approximated from small to large as Ⅴ < Ⅱ < Ⅰ < Ⅳ < Ⅲ and the stresses were approximated from small to large as Ⅴ < Ⅱ < Ⅳ < Ⅰ < Ⅲ. Figure 5 showed the von Mises stress distribution of among five groups of internal fixation devices and both bone and internal fixation. The stresses in the plate of Group Ⅰ (LPTL) were mainly concentrated at the distal end of the last two screws in the transverse row; the stresses in the plate of Group Ⅱ (LPTV) were concentrated at the points similar to those of Group Ⅰ and those of Group Ⅲ (LPOL) plate stresses were concentrated on the proximal end of the last screw and the corner of the plate; Group Ⅳ (LPOLTL) plate stresses were concentrated on the last screw, but the distal end of the two anteroposterior lag screws shared some of the stresses; and Group Ⅴ (TPP) plate stress concentrations were seen in the transverse arm proximally posteriorly lateral to the two screws and in the first screw below as well as at the plate between the three screw holes.

**TABLE 4:** Axial compression displacement of the internal fixation under three loads in five sets of fracture models.

Group	Displacement (mm)		
	250N	500N	750N
LPTL	0.27	0.54	0.81
LPTV	0.27	0.53	0.79
LPOL	0.37	0.74	1.11
LPOLTL	0.27	0.55	0.83
TPP	0.23	0.46	0.68

**TABLE 5:** Von Mises stresses of internal fixation under three loads for five sets of fracture models.

Group	Von Mises stress (MPa)		
	250N	500N	750N
LPTL	114.2	228.4	342.6
LPTV	100.44	200.89	301.34
LPOL	138.21	276.42	414.63
LPOLTL	107.22	214.45	321.68
TPP	96.07	192.14	288.21

## 4 Discussion

The choice of fixation model and surgical approach for the treatment of PTPF remain controversial. Our study evaluated the differences in strength among LCP with different screws fixation through lateral approach and distal radius plate with three screws fixation through posterior approach for the treatment of PTPF. The biomechanical test and finite element analysis showed relatively consistent results. That was PTPF with LCP and single-locking screw fixation exhibited the weakest compressive yield strength and the greatest displacement. Other four groups had good stability. The compressive yield strength of LCP with two variable angle locking screws fixation was semblable to that of posterior plate fixation. Similarly, LCP with two conventional locking screws exhibited compressive yield strength similar to that of one locking screws assisted two anteroposterior lag screws.

### 4.1 Internal fixation for posterolateral tibial plateau fracture

Through posterior approach, distal radius plate can provide multiply screws for the fixation of PL fracture fragments (Shen et al., 2019). In our study, we selected distal radius plate, utilizing two screws in the transverse arm and one in the longitudinal arm of the plate, totaling three screws for the stabilization of the fracture fragment. This fixation model (TPP) exhibited the highest level of mechanical strength for PTPF fixation. Therefore, Wang et al. (Shuaishuai et al., 2023) advocated treating PTPF through posterolateral approach. It was posited that the superior mechanical strength of the posterior plate was primarily attributed to its higher shear resistance in stabilizing fracture fragments (Giordano et al., 2022). Finite element analysis revealed that the stress on the Group TPP was concentrated between the three screws along the plate body, the shear resistance force of the plate and three screws served to distribute stress more evenly, thereby diminishing the overall stress on the internal fixation (Figures 5E, J).

Nevertheless, the posterior approach is accompanied by a potential risk of neurovascular injury, and the presence of obstructing anatomical structures, such as muscular ligaments, renders its execution comparatively more challenging (Tao et al., 2008; Cho et al., 2017; Sun et al., 2017). In contrast, the lateral approach stands out as one of the simpler and more commonly employed surgical routes, characterized by a reduced risk of vascular and nerve injury (Hu et al., 2016; Hu et al., 2020; Mao et al., 2021). In our analysis, we evaluated the number of transverse arms of the LCP utilized in stabilizing fracture fragments across various groups. We observed that with a axial load of 750 N, low support of the single-screw in the transverse arm of the plate caused further displacement exceeding 3 mm of the posterolateral (PL) fragment of fracture. This condition manifested the lowest performance in terms of compressive yield strength across all three applied axial loads, corroborating the findings reported in the existing studies by other researchers in the field (Ren et al., 2018; Sun et al., 2018; Hu et al., 2020). Finite element analysis revealed that one screw fixation led to obvious stress concentration (Figures 5C, H), which might explain fixation failure. Conversely, we observed a diminished von Mises stress of the internal fixation device in Group Ⅰ and Group Ⅱ. The dual screws shared stress together (Figures 5A, B, F, G), increasing stress dispersion. Similarly, partial stress concentrations were observed on the anteroposterior lag screws in Group IV (Figure 5D). The stresses in the plate body near the fracture line experienced a significant reduction compared to the Group of one-screw fixation (Figure 5I). This finding was consistent with the results of our biomechanical test, which demonstrated superior compression displacement, failure load, and axial stiffness in Group IV compared to Group III. The biomechanical results showed that the axial stiffness of Group Ⅰ and Group Ⅳ were very close to each other, so we believed that when two screws in the transverse arm cannot be achieved to fix the fracture fragments in clinic, we can choose to add two anterior and posterior lag screws to increase the strength of stable fixation. This fixation method was documented in earlier studies (Gao et al., 2023). Von Mises theory states that ductile materials fail when the so-called “von Mises stress” exceeds the uniaxial yield strength (Velic et al., 2021). At a loading equivalent to 300% of the body weight, the plate material exhibits a yield strength of 800 MPa (Lalwani et al., 2013). We did not find stresses in the internal fixation device that exceeded this range. This suggested that no failure risk of mechanics would be expected in the implants.

We observed similar von Mises stress distributions in both Group Ⅰ and Group Ⅱ. However, the internal fixation stresses were lower in Group Ⅱ compared to Group Ⅰ. The disparity between the two groups in the mechanical tests also reached statistical significance (*p* < 0.05). Upon analysis, we attributed this observation to the screw offset angle. It caused an increased area of screw fixation on the bone fragment resulting to a reduction in von Mises stresses.

### 4.2 Variable screw placement angle

**FIGURE 6:**
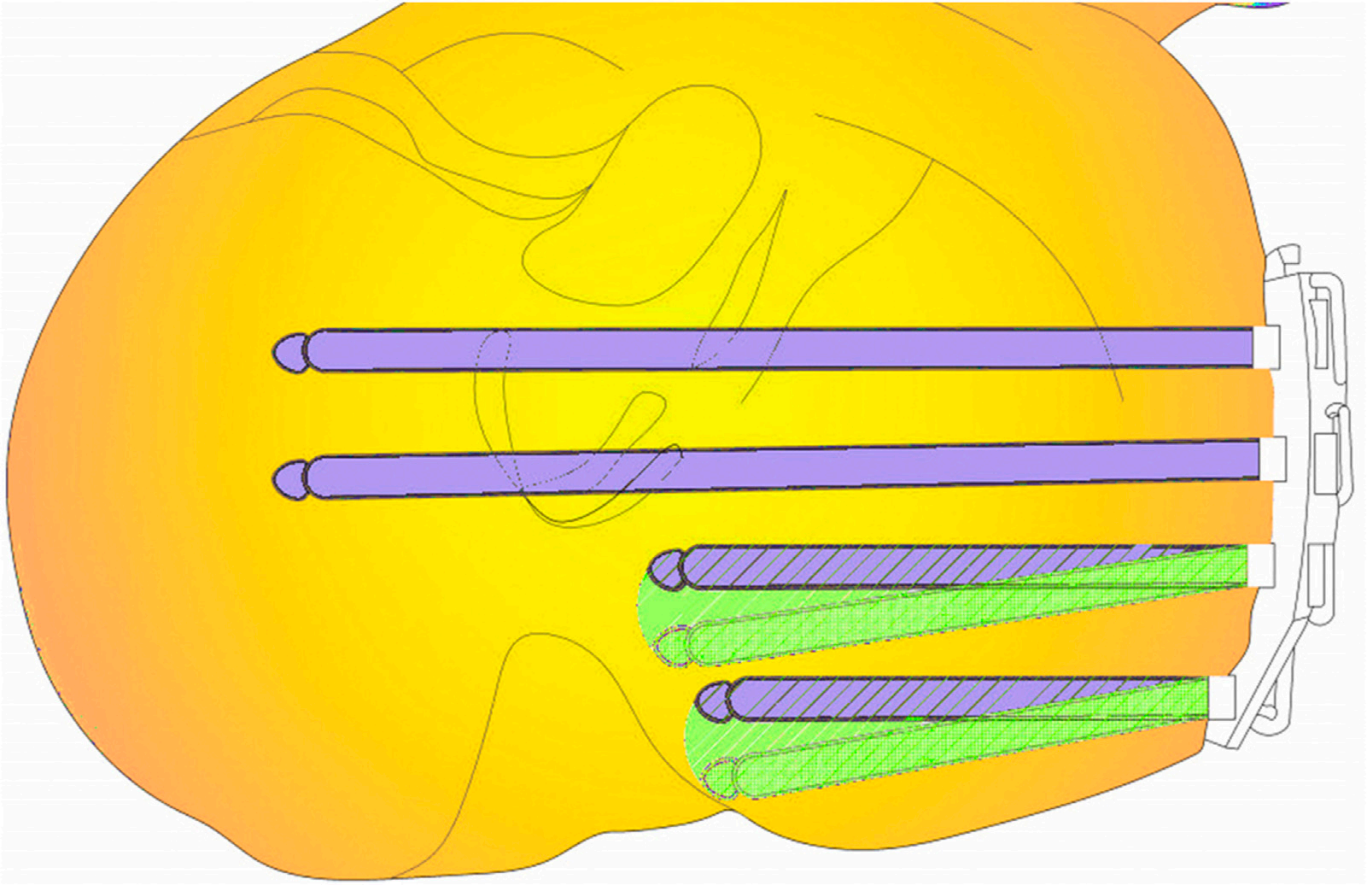
The figure **s**hows a cross section of a PL fracture. Violet color indicates conventional locking screws, green screws are variable angle locking screws, and the green range indicates an increase in fixing field.

In our study, variable angle locking screw fixation, particularly for the two posterior screws, increased the fixation area for PL fractures and concurrently enhanced fixation strength. It is important to note that large offset angles should be avoided. While Tidwell (Tidwell et al., 2016) reported decreasing fixation strength with increasing screw angle, our data contradicts this. Instead, we observed a significant increase in fixation intensity. This discrepancy might stem from the increased fixation area due to the larger offset angle. A key concern was plate screw loosening over time. Hebert-Davies (Hebert-Davies et al., 2013) found no significant reduction in torque force between the plate and screw with a multiaxial screw offset up to 10°, implying stability between the screw and implant plate. Based on these findings, we maintained a 10° offset in our experiments for stability, as illustrated in Figure 6. Consistent with previous studies (Hu et al., 2016; Jiang et al., 2018), we also ensured the length of the last variable angle screw was under 35 mm.

When the final screw was positioned with a rearward offset of 10°, we defined the offset area as a sector, represented by S′, To determine S′, the diameter of this variable angle locking screw was taken as 35 mm. Consequently, the offset area was calculated to be approximately 107 mm^2^, using the equation: $S\prime =10{}^{\circ}/360{}^{\circ}\times \pi R^{2}$ . According to Taylor et al. (Taylor et al., 2004), the load exerted on the knee joint during walking is about double an individual’s body weight. Consequently, the primary load-bearing regions of both medial and lateral platforms cover areas of approximately 389 and 363 mm^2^, respectively. These values represent 33.2% and 42.9% of the total area of the medial and lateral platforms, respectively. It is clear that the rearward offset introduced by variable angle locking screws significantly expands the PL fracture fixation area, enhancing the overall strength of the fixation.

In clinical settings, constraints such as the presence of the fibular collateral ligament or extreme posterior displacement of the PL fracture fragment can hinder the feasibility of achieving two-locking screw fixation. In such cases, the utilization of variable angle screws can fulfill the requirement for two-screw fixation of the PL fracture fragment. Importantly, our biomechanical testing and finite element analysis validated the biomechanical attributes of variable angle locking screws. When contrasted with the posterior plate, the distinction was found to lack statistical significance, despite the variable angle locking screws exhibiting inadequate mechanical strength in comparison to the posterior support plate. Given these considerations, we are inclined to believe that the use of LCP variable angle locking screws for PL fracture fixation is a viable clinical approach, with low risk of damage to the neurovascular bundle.

### 4.3 Limitations and prospects

Several limitations were acknowledged in this experiment. The study exclusively focused on the PL split fracture model, as validated models for collapsed fractures were lacking. Future experiments are planned to encompass collapsed fractures and combined cleavage-collapsed fractures. The current experimental model was constructed based on existing literature, yet the clinical spectrum of PL fractures is diverse, thus failing to encompass all fracture types. Additionally, synthetic bones were chosen for their uniformity and resemblance to human tibias in terms of dimensions and properties. This study did not use a cadaveric bone model supported by the fibula. Amirouche and Solitro et al. (Amirouche and Solitro, 2011) posit that, to date, a definitive modeling model for the knee joint has not been established. It is noteworthy that the biomechanical testing employed in this study is static and mimics an individual standing on one leg. Further studies will aim to address these limitations and provide a more comprehensive perspective on PL fracture fixation. The study was also limited by the fact that different coatings were not considered, which Patel et al. proved that there was a contribution of the coating to implant stability (Patel et al., 2015). Our study did not consider the problem of loosening that occurs when screws are inserted into the bone (Mejia et al., 2020). Additionally, the study did not explore the relationship between screw characteristics, insertion torque, and stability (Addevico et al., 2020). Other factors impacting stability such as intrannular materials (Bronsnick et al., 2015), insertion torque (Addevico et al., 2021), porous media for bone (Travascio et al., 2015), bone mechanobiology (Volz et al., 2021) were not conferred in our study.

Our objective was to assess the viability of utilizing a lateral approach for fixation, complemented by the incorporation of tension or variable angle screws, with the aim of enhancing the stability of the fractured fragment. This investigation will be pursued further in our future research endeavors.

## 5 Conclusion

Introducing variable-angle locking screws can further enhance the fixation range, with the resultant biomechanical stability similar to that of a posteriorly supported plate. It is imperative to make intraoperative adjustments to the screw arrangement to achieve the requisite mechanical strength, avoiding reliance on single screw from the plate fixing PL fracture.

## Data Availability

The original contributions presented in the study are included in the article/supplementary material, further inquiries can be directed to the corresponding authors.
